# Exogenous neuritin treatment improves survivability and functions of Schwann cells with improved outgrowth of neurons in rat diabetic neuropathy

**DOI:** 10.1111/jcmm.15627

**Published:** 2020-07-15

**Authors:** Chunhong Xi, Yingduan Zhang, Mei Yan, Qing Lv, Huan Lu, Jun Zhou, Yucheng Wang, Jianbo Li

**Affiliations:** ^1^ Endocrinology and Metabolism Department The First Affiliated Hospital of Nanjing Medical University Nanjing China; ^2^ Diabetic Neuropathy Study Group of Chinese Diabetes Society Beijing China

**Keywords:** diabetic neuropathy, neuritin, neuron outgrowth, rats, Schwann cell survival

## Abstract

Pathogenesis and treatment for diabetic neuropathy are still complex. A deficit of neurotrophic factors affecting Schwann cells is a very important cause of diabetic neuropathy. Neuritin is a newly discovered potential neurotrophic factor. In this study, we explored the effect of exogenous neuritin on survivability and functions of diabetic Schwann cells of rats with experimental diabetic neuropathy. Diabetic neuropathy was induced in rats. 12‐week diabetic rats contrasted with non‐diabetic normal rats had decreased levels of serum neuritin and slowed nerve conduction velocities (NCVs). Schwann cells isolated from these diabetic rats and cultured in high glucose showed reduced cell neuritin mRNA and protein and supernatant neuritin protein, increased apoptosis rates, increased caspase‐3 activities and progressively reduced viability. In contrast, exogenous neuritin treatment reduced apoptosis and improved viability, with elevated Bcl‐2 levels (not Bax) and decreased caspase‐3 activities. Co‐cultured with diabetic Schwann cells pre‐treated with exogenous neuritin in high glucose media, and diabetic DRG neurons showed lessened decreased neurite outgrowth and supernatant NGF concentration occurring in co‐culture of diabetic cells. Exogenous neuritin treatment ameliorated survivability and functions of diabetic Schwann cells of rats with diabetic neuropathy. Our study may provide a new mechanism and potential treatment for diabetic neuropathy.

## 1 INTRODUCTION

Peripheral neuropathy is one of most common complications of diabetes.^1^, ^2^ The exact pathogenesis of and effective treatment for diabetic neuropathy remain to be elucidated or sought.^1^, ^3^, ^4^ Enhanced polyol pathway activity, increased non‐enzymatic glycation and augmented oxidative stress are important hyperglycaemia‐related mechanisms in the development of diabetic neuropathy.^5^, ^6^, ^7^ In addition, a deficit of neurotrophic factors affecting Schwann cells (SCs) is another important cause of diabetic neuropathy.^8^, ^9^, ^10^

Demyelination of fibres constitutes one of the main pathologic characteristics of diabetic neuropathy^6^, ^11^ ^.^ SCs are essential to maintain normal structures and functions of the peripheral nervous system: formation of myelin around axons, the conduction of nervous impulses along axons, and nerve development and regeneration..^12^, ^13^ Therefore, reduced viability of SCs may significantly contribute to peripheral nerve deficits in certain conditions. Viability of SCs is compromised, leading to demyelination, as nerve fibres are exposed to unfavourable conditions including hyperglycaemia.^11^ Hyperglycaemia also results in apoptosis of SCs in vitro,^14^, ^15^ and severe hyperglycaemia‐induced apoptosis can be ameliorated by a treatment of an exogenous neurotrophic factor.^16^ So far, several neurotrophins have been found to be underproduced and involved in survival and functions of SCs in a diabetic or hyperglycaemic state.^17^, ^18^, ^19^ Apart from these, our recent findings indicate that neuritin may be a potential novel neurotrophic factor affecting SCs' viability.^20^

Neuritin, a small and highly conserved GPI‐anchored protein, is present in the central nervous system, involving neurite outgrowth and maintenance of central nervous system development.^21^, ^22^ It is also expressed in dorsal root ganglia (DRG), involving axonal regeneration, and it is deficient in DRG neurons and axons in experimental diabetic neuropathy.^23^ However, neuritin in relation to SCs in diabetic neuropathy has not been investigated. Recently, we discovered that neuritin existing in insoluble and mainly soluble (secreted) forms was expressed in SCs, and it was down‐regulated and associated with apoptosis of SCs exposed to very high glucose milieu in vitro, which were prevented by exogenous IGF‐1 (insulin‐like growth factor‐1) treatment.^20^, ^24^ In the present study, we established diabetic neuropathy model in rats as previously described^23^, ^25^ and cultured SCs and neurons isolated from the rats with and without diabetic neuropathy to explore, for the first time, the relation of compromised survival of SCs with down‐expressed neuritin to experimental diabetic neuropathy and the effect of exogenous neuritin treatment on survivability and functions of the diabetic SCs. Our study may provide a new mechanism of diabetic neuropathy and a new intervention with neuritin for diabetic peripheral neuropathy.

## 2 MATERIALS AND METHODS

### 2.1 Animals

All animals were obtained from Nanjing University Laboratory Animal Center. All experiments were conducted in accordance with Nanjing Medical University Regulations and with the Animals ACT.

### 2.2 Diabetes induction

Adult male Sprague Dawley rats, ageing 7 ± 1 w, weighing 210 ± 10 g, were used for the study. At the beginning of the experiment, they were housed at 22 ± 2°C with 12‐h alternating light/dark cycles and fed with standard laboratory rat chow. After an overnight fast, diabetes was induced in rats by a single intraperitoneal injection of STZ (Sigma, St. Louis, MO, USA) freshly dissolved in normal saline, at a dose of 55 mg/kg of bodyweight. Rats that received normal saline alone by the same route served as the control. Blood glucose was monitored from tail vein blood (OneTouch glucometer, Johnson & Johnson, New Brunswick, New Jersey, USA). STZ‐treated rats with fasting blood glucose > 16 mmol/L were accepted for the study. Rats were randomly divided into normal or diabetic groups and group‐housed with full access to food and water for 12 weeks. HbA1c (haemoglobin A1c), reflecting the mean glucose level over the past 12 weeks, was measured using high‐performance liquid chromatography (Bio‐Rad D10).Values of HbA1c were expressed as both % and mmol/mol.

### 2.3 Electrophysiological study

Each rat was anaesthetized with a peritoneal injection of pentobarbital sodium (30 ‐ 40 mg/kg). Electrophysiological studies were performed using EMG (Viking IV, Nicolet) as previously described.^19^ Briefly, the body temperature was maintained at 30°C with a heating lamp and controlled by a contact thermometer. The sciatic nerve was stimulated, and motor nerve conduction velocity (MNCV, m/s) was recorded from the first interosseous muscle of the hind paw. Sensory nerve conduction velocity (SNCV, m/s) was recorded in the digital nerve to the second toe.

### 2.4 Enzyme‐linked immunosorbent assay

Serum samples of rats or supernatant samples of SC culture were collected and processed according to assay kit manufacturer's instructions. Briefly, samples were centrifugated and added to microplates with wells coated with anti‐neuritin (Boston Biochem) or anti‐NGF (Thermo Fisher Scientific) where they were incubated at 37°C for 30 minutes. Horseradish peroxidase (HRP)* *conjugate and tetramethylbenzidine reagents were added to the plates. Absorbance (OD) was read at 450 nm on the same microplate reader (Bio‐Rad). Neuritin concentrations were measured and expressed as ng/ml in each sample, respectively. Culture‐supernatant neuritin (secreted) or NGF concentrations were expressed as ng/g of total cellular protein using the Bradford method.

### 2.5 Histochemical immunostaining

Sciatic nerves were isolated from normal and diabetic rats, respectively, at the week 12 of the experiment. A routine histochemical procedure was conducted as previously described.^24^ Briefly, 0.5‐cm‐long sciatic nerves were paraffin‐embedded, cut into 5‐µm‐thick longitudinal sections and further processed. SCs were identified by immunostaining using rabbit anti‐S‐100 (1:100, antibody specific to SCs) (Proteintech). Neuritin was identified using immunostaining with goat anti‐rat neuritin antibody (1;100, Santa Cruz, Dallas, Texas, USA). Slides were washed and incubated with DyLight 549‐conjugated goat anti‐rabbit antibody (1:100, Proteintech) or fluorescein isothiocyanate (FITC)‐conjugated donkey anti‐goat antibody (1:100, Proteintech), respectively. Each picture was viewed using a Leica fluorescence microscope (Leica MZ FL b, Leica Microsystems).

### 2.6 SC culture and treatment

Schwann cells were isolated and purified from the sciatic nerves of these normal control or diabetic rats as previously described.^20^, ^24^ Briefly, after whole epineurium was stripped off, epineurium‐free nerve tissue was collected and transferred to petri dishes where the dissociation solution was added (DMEM, 10% FCS, 1% pen/strep, 0.125% collagenase, and 1.25 U mL/1 dispase) and incubated for 20 hours at 37°C, and the dissociated tissues were transferred and separated into single cells that were centrifuged, resuspended and seeded. Cells were initially plated on 6‐cm petri dishes coated with 0.001% poly‐L‐lysine (Sigma) and 5 µg/mL laminin (Gibco‐BRL, New York, NY, USA), containing the growth medium (DMEM/ F12, 10% foetal bovine serum (Hyclone), 20 mg/L bovine pituitary extract (Science cell, Carlsbad, CA, USA), 2 µmol/L forskolin (Sigma) and 5 µg/mL cytosine arabinoside (Sigma). SCs were mainly identified by high levels of immunostaining using rabbit anti‐S‐100 (1:100, antibody specific to SCs) (Proteintech). Additional DAPI (4′,6‐diamidino‐2‐phenylindole) (SouthernBiotech) staining was used to measure purity of SCs in culture. Neuritin was identified using immunostaining with goat anti‐rat neuritin antibody (1;100, Santa Cruz, Dallas, Texas, USA). The two proteins were visualized using DyLight 549 or 488‐conjugated goat anti‐rabit antibody (1:100, Proteintech) or fluorescein isothiocyanate (FITC)‐conjugated donkey anti‐goat antibody (1:100, Proteintech), respectively. After confluency in the growth medium and 3 passages, SCs (purity, 90%) were cultured for experiments. Experimental media were based on the medium: DMEM (low glucose)/Ham's F‐12 1:1 (Gibco‐BRL), 2 µmol/L forskolin (Sigma‐Aldrich), transferrin (10 mg/mL), putrescine (10 mmol/L) and progesterone (20 nmol/L). To this serum‐free medium, glucose was added: 5.6 mmol/L glucose mimicking normal glucose condition and 25 mmol/L glucose mimicking high glucose condition, respectively. Exogenous recombinant neuritin (5 or 10 ng/mL) (PeproTech) was used to treat SCs in certain conditions. For each experiment, SCs grown in growth media were first washed with PBS (Gibco) and cultured in conditioned serum‐free media for certain length of time. Experimental SCs were basically grouped into: (a) NSC, normal control SCs, isolated from normal rats and cultured in normal glucose (5.6 mmol/L); (b) DSC, diabetic SCs, isolated from diabetic rats and cultured in high glucose (25 mmol/L); and (c) DSC + NEU, diabetic SCs treated with exogenous neuritin.

### 2.7 Q‐PCR measurement

Q‐PCR measurement was conducted as previously described.^20^, ^24^ Briefly, following reverse transcription to cDNA, the PCR reactions were performed according to standard procedures with SYBR Premix Ex Taq (Hercules, CA, USA). Amplifications of specific cDNA were carried out using the following primers: for neuritin: forward primer, 5′TGAGAGCAGCAGGCAAGTG3′; reverse primer, 5′CGGTCTTGATGTTCGTCTTGTC3′.

The amplification profile was initial denaturation at 94°C/3 minutes, 35 cycles at 94°C/30 seconds, 60°C/30 seconds and 72°C/1 minutes, and a final incubation at 72°C/5 minutes. Sequence analysis was performed using ABI PRISM 310 (PE ABI, NY, USA). Data were expressed as relative mRNA levels calculated by the 2[−ΔΔC(T)] method.

### 2.8 Western blotting

Western blotting was conducted largely as previously reported.^20^, ^24^ Briefly, SCs were collected and homogenized in ice‐cold lysis buffer. Proteins (60 µg each sample) were separated by 0.1% SDS‐PAGE (Amresco) and electrophoretically transferred to nitrocellulose (Roche). Proteins were incubated in Tris‐buffered saline (5% BSA (Sigma), 5% casein (Sigma), and 0.05% Tween‐20) at 37°C for 1 hour, and then incubated with anti‐neuritin (1:300; R&D systems), anti‐β actin (1:5000; Sigma), anti‐Bcl‐2 (1:1000; CST, Beverly, Massachusetts, USA) or anti‐Bax (1:1000; CST) overnight at 4°C, respectively. Blots were washed and incubated with horseradish peroxidase‐conjugated anti‐goat IgG (1:5000, Santa Cruz, CA) at 37°C for 1 hour. The protein complexes were quantified using densitometric analysis (LeicaQ570C, Solms, Germany). Data were expressed as neuritin, Bcl‐2 or Bax to β‐actin ratios (arbitrary units), respectively.

### 2.9 Annexin V/PI apoptosis assay

As previously described,^20^, ^24^ Apoptosis Assay Kit (Invitrogen) for flow cytometry was used to analyse apoptosis. Briefly, following the manufacturer's instructions, SCs were washed, supernatant was discarded, and cells were resuspended with Annexin‐binding buffer. Cells were stained with Alexa Fluor 488 Annexin V and propidium iodide (PI, 1 µg/mL) and incubated at room temperature for 15 minutes. The stained cells were analysed by measuring the fluorescence emission at 530 nm and 575 nm using 488 nm excitation. The percentage of cell membrane phosphatidylserine residues labelled with Annexin V was measured using FACS Aria™ cell sorter (BD, Franklin Lakes, NJ, USA). For each measurement, 10 000 cells were counted. Data were expressed as the percentage of Annexin V stained‐positive cells relative to the total cell population in each group.

### 2.10 TUNEL apoptosis assay

Fluorescein isothiocyanate–labelled TUNEL assay (Roche) was conducted as previously described.^20^, ^24^ Briefly, firstly, SCs on chamber slides were fixed with 4% paraformaldehyde, and incubated with fluorescein isothiocyanate–labelled TUNEL, at 37°C for 1 hour in the dark. The slides were counterstained with propidium iodide for 15 minutes on ice and treated with terminal deoxynucleotidyl transferase or without (but with the same volume of label solution), respectively. Positive controls were treated with DNase I before labelling. Samples were studied using ImageXpress Velos Laser Scanning Cytometer (Molecular Devices). Data were expressed as the percentage of TUNEL‐positive cells relative to the total cell population in each group.

### 2.11 Cell Counting Kit‐8 (CCK8) viability assay

Schwann cells were seeded and grown in 96‐well plates (2 × 10^3^ cells/well) for 24 hours. After treatment, viability of cells was measured using the Cell Counting Kit‐8 (CCK8) assay (DOJINDO LABORATORIES, Mashikimachi, kamimashiki gun Kumamoto, Japan) following the manufacturer's instructions. Briefly, 10 µL CCK8 reagents were added to each well and cells were incubated at 37°C for 60 minutes. The absorbance (optical density) at 450 nm was measured, reflecting the viability of cells. Data were expressed as per cent changes in the viability of samples over controls.

### 2.12 Caspase fluorometric assay

Schwann cells were collected, washed and lysed in an ice‐cold lysis buffer. The assay was conducted according to the caspase‐3 fluorometric assay kit manufacturer's instructions (BioVision), measuring fluorescence emitted by the cleavage of a synthetic fluorescent substrate. Absorbance was read on a microplate reader (Model 680, Bio‐Rad) at 405 nm. Data were expressed as fold increases in caspase activities of apoptotic samples over controls.

### 2.13 Sensory neuron culture

Sensory neurons were isolated from DRGs dissected out from spinal segments L4 and L5 of rats.^23^ Neurons were suspended in 35‐mm petri dishes pre‐coated with 0.025 μg/μL rat tail collagen (Type I) (St. Louis, Missouri, United States) with Neurobasal‐A Media supplemented with B‐27 Supplement (50:1) (Gibco, Carlsbad, California) (containing 1 mmol/L l‐glutamine, 4 mmol/L 5‐FUDR). The purity of neurons in culture was nearly 90%.

### 2.14 Co‐culture of SCs and neurons

Isolated SCs were cultured as above and seeded at a density of 2 × 10^5^/mL into 35‐mm petri dishes with the growth media. Following formation of a single layer of SCs, SCs were washed with PBS, treated with exogenous neuritin for 48 hours, re‐washed with PBS and cultured in experimental media. Isolated sensory neurons were seeded over the SCs on the same petri dishes. Co‐culture was divided into three groups: (a) normal control neurons and NSC, isolated from normal rats and cultured in normal glucose (5.6 mmol/L); (b) diabetic neurons and DSC, isolated from diabetic rats and cultured in high glucose (25 mmol/L); and (c) diabetic neurons and DSC + NEUpre (diabetic SCs pre‐treated with exogenous neuritin), cultured in high glucose (25 mmol/L).

### 2.15 Immunocytochemistry and neurite outgrowth analysis

Co‐cultured cells were fixed with ice‐cold 4% paraformaldehyde for 15 minutes, washed three times with PBS, incubated with 0.25% Triton X‐100 for 10 minutes and then incubated with 1% BSA for 30 minutes. Fixed cells were incubated with mouse anti‐(III) tubulin (1:1000, Sigma) or anti‐NF‐200 antibody (1:100, Abcam) at 4°C overnight. Cells were washed and incubated with fluorescein isothiocyanate (FITC)‐conjugated donkey antimouse (1:100, Proteintech) for 1 hours at room temperature. Immunofluorescence was viewed using a Leica fluorescence microscope and images were analysed using IMAGE J software. Images of 30 neurons were randomly selected from fields of view for each experimental condition. The average length of neurite outgrowth and the length of the longest neurite of each neuron (distance from the cell body) were calculated according to a method previously described.^23^

### 2.16 Statistical analysis

All above measurements were repeated 3 times in each independent experiment. Data were expressed as mean ± SE of 3‐6 independent experiments. One‐way ANOVA for multiple comparisons of quantitative mRNA and proteins, and post hoc test and chi‐square test for percentage comparisons of apoptosis rates were conducted. *P* value < .05 was considered to be significant.

## 3 RESULTS

### 3.1 Increased BG, decreased serum neuritin and slowed NCVs in diabetic rats

Diabetic rats showed polydipsia, polyuria, polyphagia, muscle wasting and progressive loss of bodyweight over 12 weeks of diabetic course. During this period, diabetic rats had an obviously elevated level of blood glucose that was monitored stable and HbA1c that reflected mean blood glucose concentrations over the past 12 weeks. In these diabetic rats, increasingly decreased levels of serum neuritin and gradually slowed velocities of both motor and sensory nerve conduction were observed, which started at week 2 of the experiment (data not shown). These changes in diabetic rats were contrasted with non‐diabetic normal rats, in particular at week 12 of diabetic course (Table 1).

**TABLE 1:** BG, HbA1c, BW, serum neuritin and NCVs in rats at week 12

	NC (n = 15)	DM (n = 12)	*P* value
BG (mmol/L)	5.7 ± 0.8	24.8 ± 4.2	<0.0001
HbA1c (%)(mmol/mol)	5.1 ± 0.6 (32 ± 4)	14.5 ± 3.7 (135 ± 17)	<0.0001
BW (g)	327.5 ± 34.8	187.4 ± 18.6	<0.0001
SNEU (ng/mL)	2.8 ± 0.3	1.9 ± 0.2	<0.01
SNCV (m/s)	28.7 ± 2.1	18.1 ± 2.6	<0.005
MNCV (m/s)	22.3 ± 3.0	15.1 ± 2.5	<0.01

Note

Data were expressed as mean ± SD *P* value, DM vs NC.

Abbreviations: BG, blood glucose; BW, bodyweight; DM, diabetic rats; HbA1c, hemoglobin A1c; MNCV, a motor nerve conduction velocity; NC, normal control rats; SNEU, serum neuritin; SNVC, a sensory nerve conduction velocity.

### 3.2 Decreased neuritin expression, increased apoptosis and decreased viability of diabetic SCs

**FIGURE 1:**
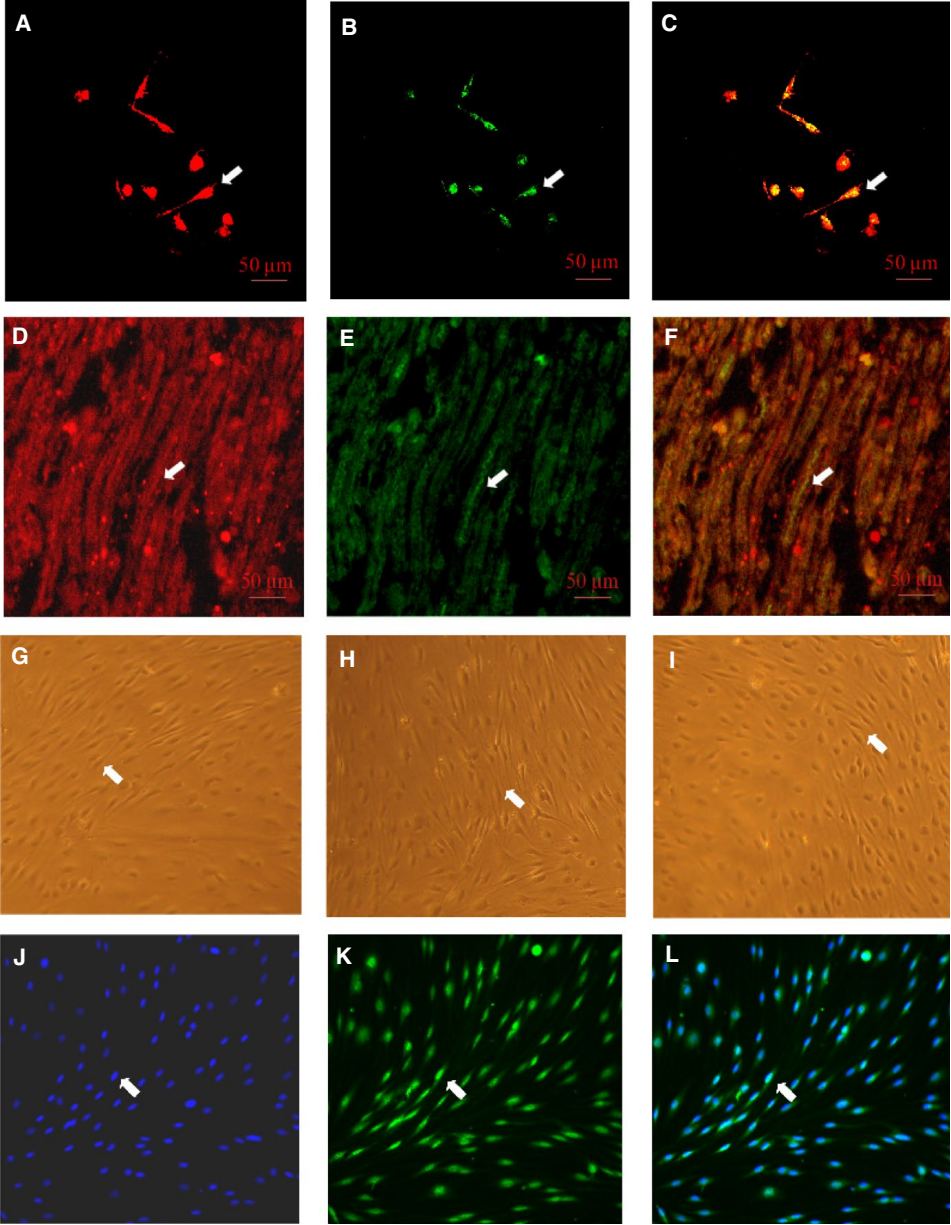
Representative neuritin immunostaining in Schwann cells isolated from or within sciatic nerves of 12‐wk duration diabetic rats. Schwann cells were identified by high levels of immunostaining using anti‐S‐100. Neuritn was identified using immunostaining with anti‐neuritin antibody. Bar = 50 μm. For cytochemical immunostaining: A, S‐100 in red (DyLight 549); B, neuritin in green (FITC); C, the A and B emerged shown in yellow. For histochemical immunostaining: D, S‐100 in red (DyLight 549); E, neuritin in green (FITC); F, the D and E emerged shown in yellow. G, H or I: Phase‐contrast micrographic Schwann cells (spindle shaped, at 10× magnification) at the passage 1, 2 or 3 in cultures, respectively; J, K or L: Schwann cell staining (10× magnification) for purity measurement in culture at the passage 3, DAPI staining in blue, S‐100 staining in green (DyLight 488) or the two staining emerged in culture indicating the Schwann cell purity (90%), respectively. A typical example was shown in each micrograph with a white arrow

**FIGURE 2:**
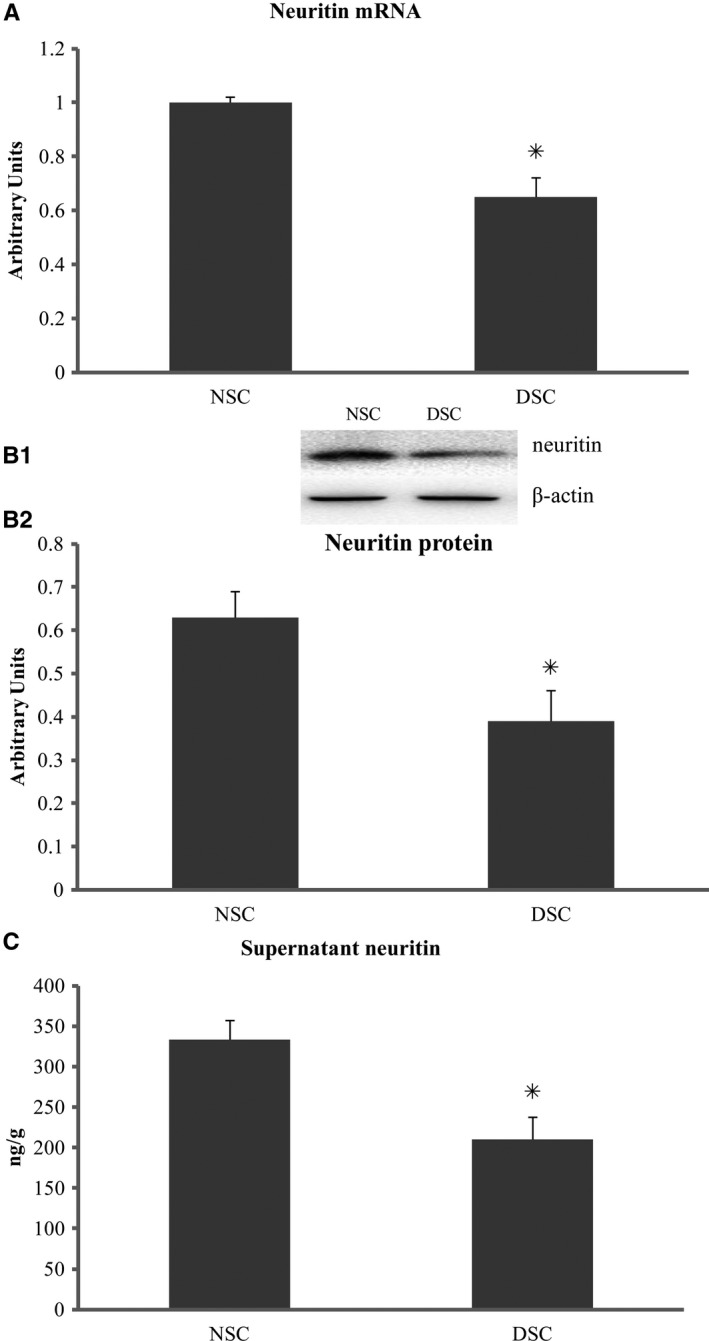
Neuritin expression in Schwann cells. Isolated from sciatic nerves of diabetic rats with decreased serum neuritin and slowed NCVs, Schwann cells in culture for 48 h showed decreased cell neuritin mRNA (A) and protein (B), and culture‐supernatant neuritin concentrations (C) in contrast to those from normal control rats. NSC, normal control Schwann cells, isolated from normal rats and cultured in normal glucose (5.6 mmol/L). DSC, diabetic Schwann cells, isolated from diabetic rats and cultured in high glucose (25 mmol/L). B1: An example of neuritin and β‐actin bands using Western blotting. A, B2, and C: DSC vs NSC, *P < .01, respectively. Data were expressed as mean ± SE of 6 independent experiments

**FIGURE 3:**
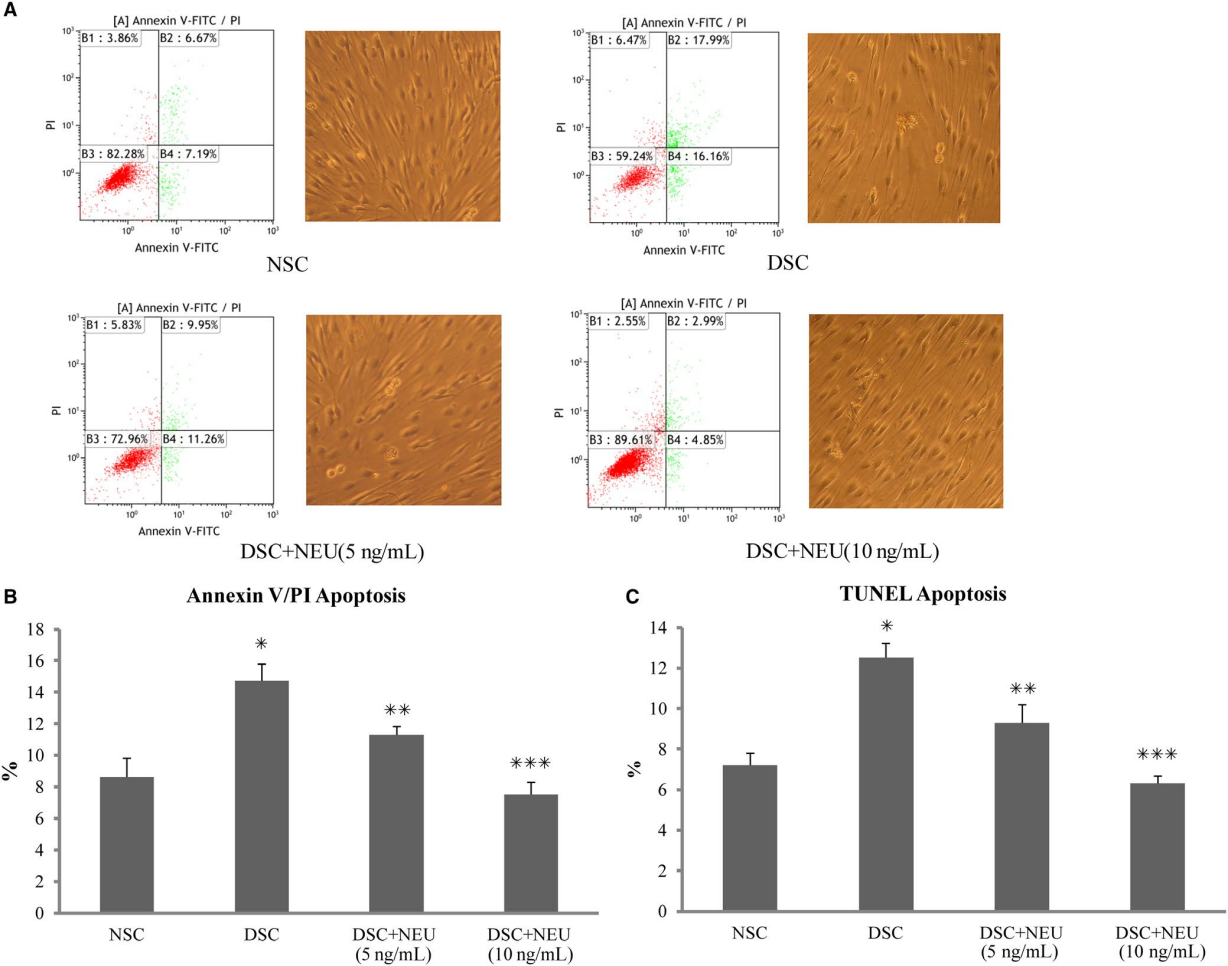
Apoptosis in Schwann cells. Isolated from sciatic nerves of diabetic rats with decreased serum neuritin and slowed NCVs, Schwann cells in culture for 48 h showed increased apoptosis rates using Annexin V/PI assay (A and B) and TUNNEL assay (C) in contrast to those from normal control rats, respectively. These diabetic Schwann cells in culture treated with exogenous neuritin had reduced apoptosis rates. The apoptosis rates decreased with an increasing dose of neuritin treatment (from 5 to 10 ng/mL) (A, B, and C). NSC, normal control Schwann cells, isolated from normal rats and cultured in normal glucose (5.6 mmol/L). DSC, diabetic Schwann cells, isolated from diabetic rats and cultured in high glucose (25 mmol/L). DSC + NEU, DSC treated with exogenous neuritin (5 or 10 ng/mL). A: The examples of apoptotic Schwann cells using Annexin V/PI assay and phase‐contrast pictures of Schwann cells (20 × magnification), with the right lower quadrant showing early apoptotic cells and the left lower quadrant showing viable cells in each graph, respectively. B and C: DSC vs NC, *P < .01; DSC + NEU vs DSC, **P < .05, ***P < .01, respectively. No statistical differences were found between NSC and DSC + NEU 10 ng/mL. Data were expressed as mean ± SE of 6 independent experiments

**FIGURE 4:**
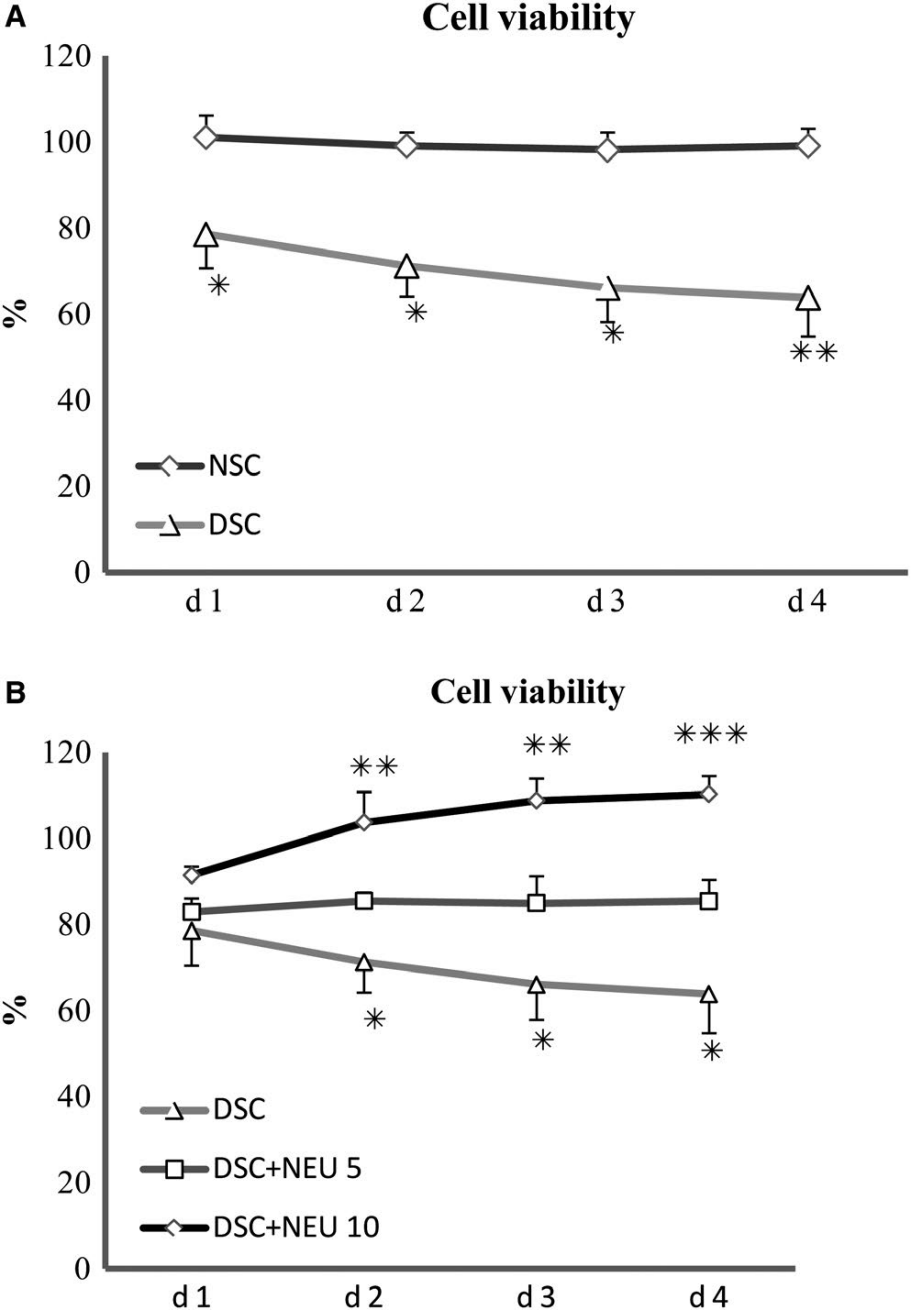
Viability of Schwann cells. Isolated from sciatic nerves of diabetic rats with decreased serum neuritin and slowed NCVs, Schwann cells showed progressively decreased relative viability (%) over 4 days in culture in contrast to those from normal control rats (A). These diabetic Schwann cells in culture treated with exogenous neuritin had reduced compromised viability using CCK‐8. The viability increased with an increasing dose of neuritin treatment (from 5 to 10 ng/mL) (B). NSC, normal control Schwann cells, isolated from normal rats and cultured in normal glucose (5.6 mmol/L). DSC, diabetic Schwann cells, isolated from diabetic rats and cultured in high glucose (25 mmol/L). DSC + NEU, DSC treated with exogenous neuritin (5 or 10 ng/mL). A, DSC vs NSC, *P < .05, **P < .005, respectively. B, DSC + NEU (5 ng/mL) vs DSC, *P < .05; DSC + NEU (10 ng/mL) vs DSC, **P < .01, ***P < .005, respectively. No statistical differences were found between NSC and DSC + NEU 10 ng/mL. Data were expressed as mean ± SE of 6 independent experiments

Neuritin was localized using immunostaining in the cytoplasms of SCs isolated from or within sciatic nerves of rats (Figure 1) at week 12 of the experiment. SCs, which were isolated the diabetic rats that demonstrated decreased serum neuritin concentrations and slowed nerve conduction velocities and then cultured for 48 hours in high glucose media, were found to have decreased amounts of cell neuritin mRNA (Figure 2A) and protein detected from cell extracts (Figure 2B), and lowered neuritin concentrations detected from culture supernatants reflecting soluble or secreted portion of neuritin (Figure 2C), compared to SCs isolated from normal age‐controlled rats and cultured in normal glucose. In a small percentage (nearly 14%) of these diabetic SCs, early apoptotic changes were found using Annexin V/PI and TUNEL assays, and this was obvious if compared to non‐diabetic normal cells cultured in a normal glucose condition (Figure 3A,C). In addition, a dynamic assessment of the viability (%) of SCs using CCK8, reflecting the number of viable cells and the ability of cell proliferation, showed that diabetic SCs had lower and progressively lower viability, from nearly 80% to 60%, compared to normal SCs over 4 days from day 1 to day 4 in respective cultures (Figure 4A).

### 3.3 Improved apoptosis and viability of diabetic SCs treated with exogenous neuritin

Schwann cells isolated from these 12‐week diabetic rats with serum neuritin concentrations and slowed nerve conduction velocities were treated with exogenous neuritin for 48 hours in high glucose culture. These neuritin‐treated diabetic SCs showed decreased apoptosis rates by nearly 25%, compared to untreated diabetic SCs cultured in high glucose condition (Figure 3B,C). Consistently, the viability (%) of these neuritin‐treated diabetic SCs was shown gradually increased from 20% to 40%, compared to untreated SCs over 4 days from day 1 to day 4 in respective cultures, although the viability was not obviously different between them at day 1 and the obvious difference started to be shown from day 2 (Figure 4B). In addition, apoptosis and viability of SCs were increasingly improved with an incrementing dose of exogenous neuritin treatment. In general, the extent of the effect of 10 ng/mL exogenous neuritin was greater than 5 ng/mL exogenous neuritin (Figures 3B,C, and 4B).

### 3.4 Depressed caspase‐3 and elevated Bcl‐2 in diabetic SCs treated with exogenous neuritin

**FIGURE 5:**
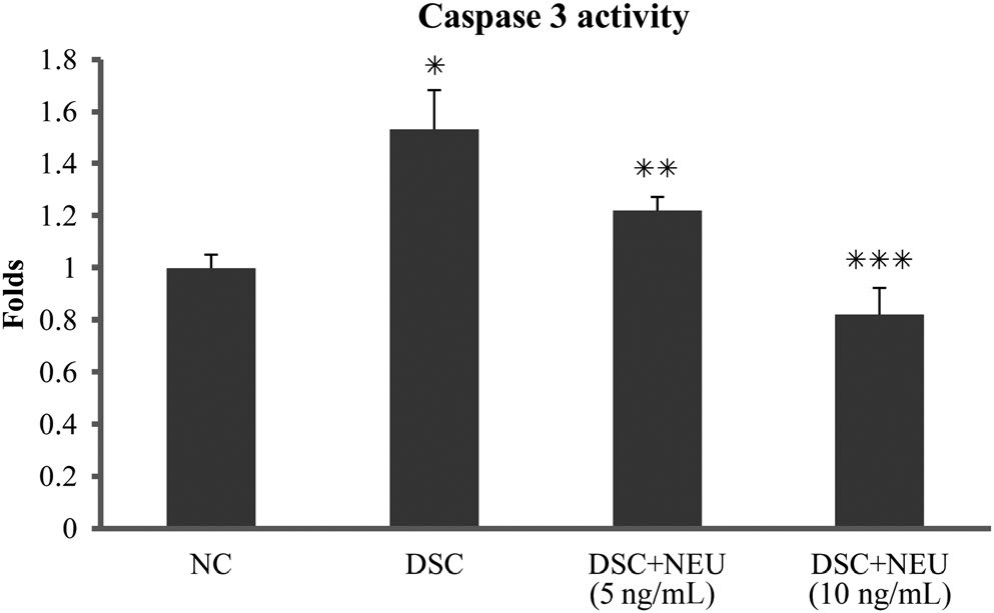
Caspase‐3 activity of Schwann cells. Isolated from sciatic nerves of diabetic rats with decreased serum neuritin and slowed NCVs, Schwann cells in culture for 48 h showed an increased relative activity (folds) of caspase‐3 in contrast to those from normal control rats. These diabetic Schwann cells in culture treated with exogenous neuritin had a decreased activity of caspase‐3 compared to without. Caspase‐3 activity decreased with an increasing dose of neuritin treatment (from 5 to 10 ng/mL). NSC, normal control Schwann cells, isolated from normal rats and cultured in normal glucose (5.6 mmol/L). DSC, diabetic Schwann cells, isolated from diabetic rats and cultured in high glucose (25 mmol/L). DSC + NEU, DSC treated with exogenous neuritin (5 or 10 ng/mL). *: DSC vs NSC, *P* < .01. **: DSC + NEU vs DSC, *P* < .05. ***: DSC + NEU vs DSC, *P* < .01. No statistical differences were found between NSC and DSC + NEU 10 ng/mL. Data were expressed as mean ± SE of 6 independent experiments

**FIGURE 6:**
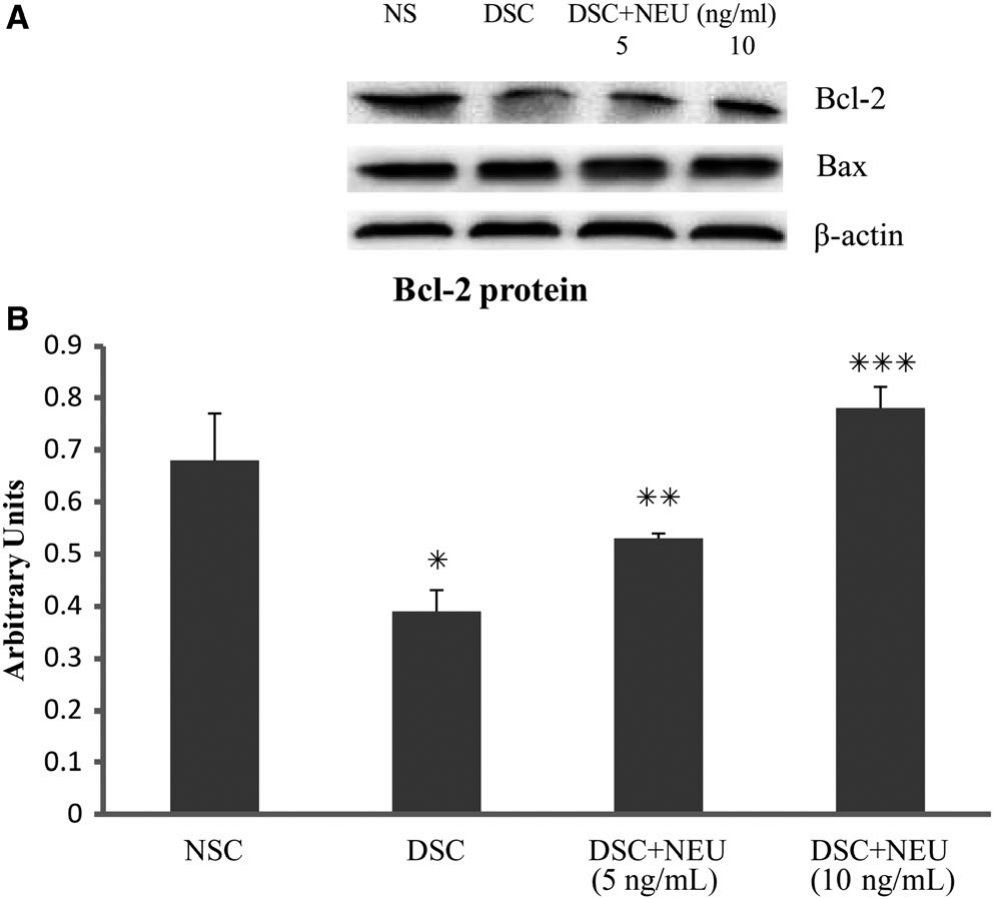
Bcl‐2 and Bax of Schwann cells. Isolated from sciatic nerves of diabetic rats with decreased serum neuritin and slowed NCVs, Schwann cells in culture for 48 h showed decreased Bcl‐2 contents in contrast to those from normal control rats (A and B). These diabetic Schwann cells in culture treated with exogenous neuritin had less reduction of Bcl‐2 compared to without. Bcl‐2 increased with an increasing dose of neuritin treatment (from 5 to 10 ng/mL). Bax did not have these changes in respective cultures (A and B). NSC, normal control Schwann cells, isolated from normal rats and cultured in normal glucose (5.6 mmol/L). DSC, diabetic Schwann cells, isolated from diabetic rats and cultured in high glucose (25 mmol/L); DSC + NEU, DSC treated with exogenous neuritin (5 or 10 ng/mL). A, The example of Bcl‐2, Bax and β‐actin bands using Western blotting. B, DSC vs NSC, *P < .01; DSC + NEU vs DSC, **P < .05, ***P < .01, respectively. No statistical differences were found between NSC and DSC + NEU 10 ng/mL. Data were expressed as mean ± SE of 6 independent experiments

These diabetic SCs that were treated with exogenous neuritin for 48 hours in high glucose culture showed decreased activities of caspase‐3 and elevated levels of Bcl‐2 protein compared to untreated diabetic SCs (Figures 5 and 6). Like its effects on apoptosis and viability of SCs, exogenous neuritin treatment depressed the activity of caspase‐3 and increased the level of Bcl‐2 (not Bax) in a dose‐dependent manner. In contrast, untreated diabetic SCs showed an increased activity of caspase‐3 and a decreased level of Bcl‐2 protein (not Bax) compared to non‐diabetic normal SCs.

### 3.5 Improved neurite outgrowth of neurons in co‐culture with diabetic SCs pre‐treated with exogenous neuritin

**FIGURE 7:**
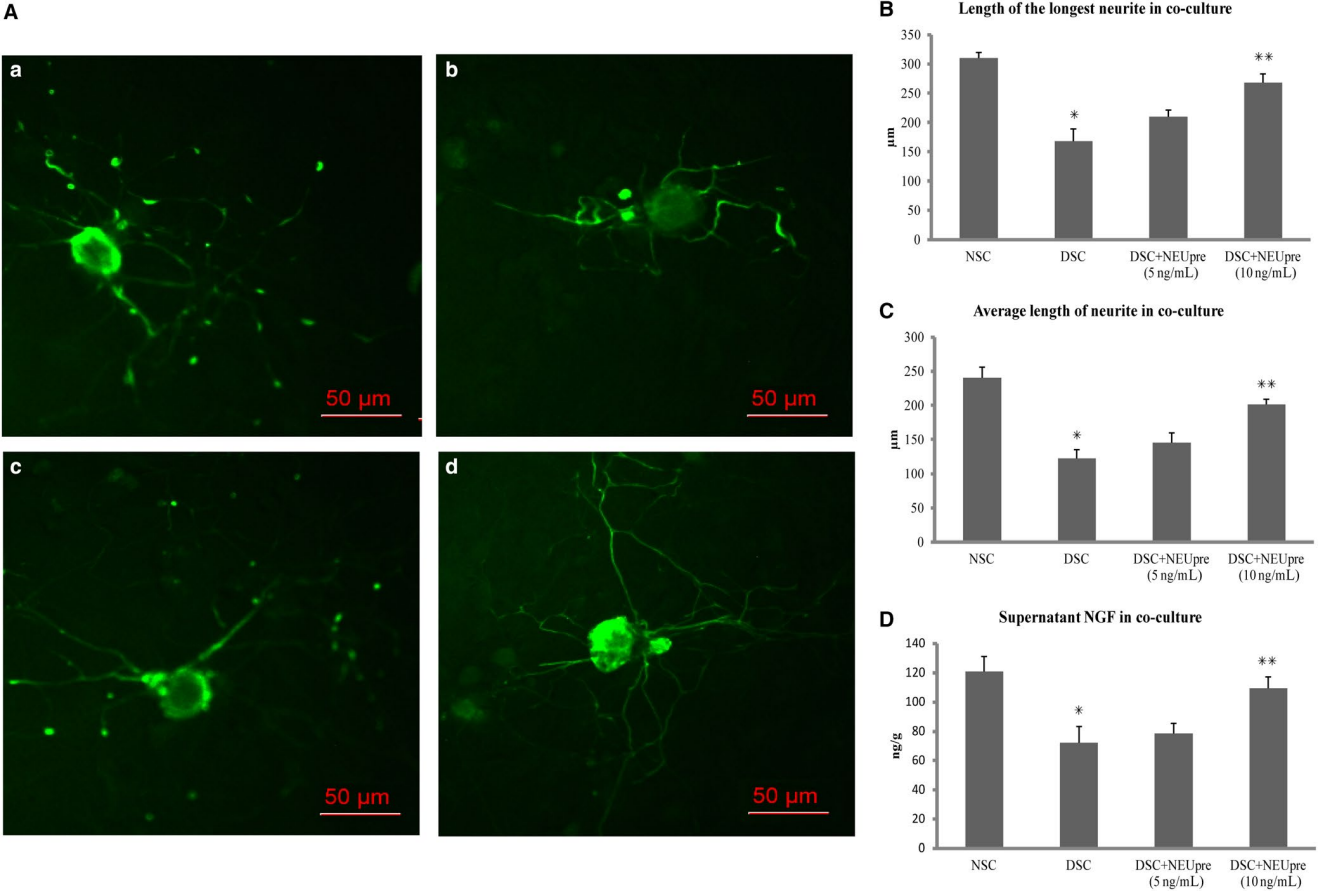
Neurite outgrowth of neurons and supernatant NGF in co‐culture with Schwann cells. A, Examples of neurites stained using mouse anti‐NF‐200 antibody with immunofluorescence viewed (shown in green [FITC]) in co‐culture with Schwann cells. Bar = 50 μm. a, normal control neurons and NSC co‐cultured in normal glucose (5.6 mmol/L); b, diabetic neurons and DSC co‐cultured in high glucose (25 mmol/L); c, DSC pre‐treated with exogenous neuritin (5 or 10 ng/mL) (DSC + NEUpre) and diabetic neurons co‐cultured in high glucose (25 mmol/L). B, C and D: Isolated from diabetic rats with decreased serum neuritin and slowed NCVs and co‐cultured with diabetic Schwann cells (DSC) for 48 hours in high glucose media, DRG neurons showed a reduction of both longest neurite outgrowth (B) and average length of neurite outgrowth (C), and supernatant NGF concentration (D), compared to those DRG neurons and Schwann cells (NSC) from normal control rats. Diabetic DRG neurons co‐cultured with diabetic Schwann cells pre‐treated with exogenous neuritin (DSC + NEUpre) had less reduction of neurite outgrowth (B and C) and supernatant NGF concentration (D), compared to without. These parameters increased with an increasing dose of neuritin treatment (from 5 to 10 ng/mL). *: DSC vs NSC, *P* < .01. **: DSC + NEUpre vs DSC, *P* < .05. Data were expressed as mean ± SE of 6 independent experiments

Dorsal root ganglia neurons, which were isolated from diabetic rats with decreased serum neuritin and slowed NCVs and co‐cultured with diabetic SCs for 48 hours in high glucose media, showed a reduction of both longest neurite outgrowth (Figure 7A,B) and average length of neurite outgrowth (Figure 7C), and supernatant NGF concentration (Figure 7D), compared to those DRG neurons co‐cultured with SCs from normal control rats in normal glucose media. In contrast, these diabetic DRG neurons co‐cultured with diabetic SCs that were pre‐treated for 48 hours with exogenous neuritin before co‐culture had less reduction of two measurements of neurite outgrowth (Figure 7B,C) and supernatant NGF concentration (Figure 7D). These parameters increased with an increasing dose of neuritin treatment (from 5 to 10 ng/mL), although 5 ng/mL of exogenous neuritin treatment did not show a significant difference from without.

## 4 DISCUSSION

In this study, we demonstrated decreased serum neuritin concentrations in established neuropathy with obviously slowed nerve conduction velocities in rats with 12‐week diabetic duration. Our previous study found neuritin was produced from SCs and present as a small molecule (10‐15 kD) in insoluble and mainly soluble (secreted) forms, with the latter detected in cell‐culture supernatants.^21^ Therefore, this small and soluble portion secreted from SCs can be one of sources of neuritin in extracellular fluids including sera. In our present study, SCs, which were isolated from diabetic rats with decreased serum neuritin concentrations and cultured in high glucose mimicking in vivo hyperglycaemic condition, had decreased culture‐supernatant neuritin concentrations due to down‐expressed neuritin. These indicate that hyperglycaemia‐induced neuritin down‐expression in SCs contributes, at least partially, to reduced serum neuritin concentrations, which may be associated with slowed nerve conduction velocities, in rats with diabetic neuropathy. More importantly, the decreased neuritin production is associated with reduced survival of SCs. In one separate study (unpublished), we used lentivirus transfection to silence neuritin gene of SCs isolated from normal rats, leading to nearly 70% drop of neuritin production with nearly 60% rise of apoptosis rate.^20^ The present study showed that diabetic SCs with down‐expressed neuritin, which manifested as decreased cell neuritin mRNA and protein and supernatant neuritin concentrations, had significantly increased apoptosis rates and progressively decreased viability in the dynamic observation, and the decreased survivability of these SCs was alleviated by exogenous neuritin treatment. Therefore, neuritin is vital to survival of SCs, diabetic or hyperglycaemic conditions can induce neuritin down‐expression in SCs, and underproduction of neuritin including soluble neuritin secreted to extracellular fluids contributes, to some extent, to compromised survival of SCs of rats with diabetic neuropathy. Furthermore, we observed that diabetic SCs with increased viability after exogenous neuritin pre‐treatment reduced compromised neurite outgrowth of co‐cultured DRG neurons from diabetic rats. These findings on SCs would add the importance of neuritin to development of and treatment for diabetic neuropathy, in addition to neuritin expressed by DRG neurons previously reported by others.^22^

In the intervention, we used exogenous neuritin mimicking replacement treatment in an auto‐ or paracrine pattern, at an incremented dose, to mitigate decreased survival of diabetic SCs in the aspects of apoptosis and viability and thus improve SC‐associated neurite outgrowth of neurons (co‐culture). Interestingly, we did not observe this significant effect of exogenous neuritin if administrated at 2.5 ng/mL (data not shown), an approximate level of diabetes or hyperglycaemia‐depleted neuritin concentrations in sera or cell‐culture supernatants of rats in our experiment. However, exogenous neuritin gradually increased its effect if given at 5 ng/mL to 10 ng/mL, exceedingly above the depletion level. It is probably explained that diabetic neuropathy is a chronic complication^1^, ^4^, ^5^, ^6^; therefore, SCs may lose their survivability as a result of the cumulative effect of long standing hyperglycaemia marked by elevated HbA1c, and exogenous neuritin, if given for a certain period and as a single treatment, needs to be dosed up to certain extent to revive the poorly viable diabetic SCs with early apoptosis and promote cell proliferation as shown in our experiment. Similarly, dosed‐up exogenous neuritin, for instance, 10 ng/mL in our experiment, is needed to produce more viable SCs to reverse compromised neurite outgrowth of neurons exposed to long duration of diabetes. We further investigated the action pathway of exogenous neuritin. In SCs alone culture, exogenous neuritin promoted anti‐apoptotic Bcl‐2 production not pro‐apoptotic Bax and depressed caspase‐3 activity, without affecting glucose levels throughout our experiment (data not shown), indicating that exogenous neuritin inhibits apoptosis of diabetic SCs at least through enhancing anti‐apoptotic pathway. After SCs were treated with exogenous neuritin, viable SCs increased with increased production of NGF, and neurite outgrowth of co‐cultured DRG neurons from diabetic rats was obviously improved, indicating that at least NGF, a major enhancer of neurite outgrowth of neurons, is an important element in the crosstalk.

Peripheral neuropathy is a common complication associated with diabetes, affecting millions of people around the world.^2^ Unfortunately, pathogenesis of and treatment for diabetic neuropathy are still complex. Demyelination is one feature of peripheral diabetic neuropathy,^6^, ^11^ which is also characterized by remyelination, if properly treated, particularly in the early stage.^6^, ^25^, ^26^ Since SCs affect many important aspects of peripheral nerve biology,^12^, ^13^ poor viability of SCs unavoidably affects nerve functions. Our study, for the first time, demonstrated the role of neuritin‐related survivability of SCs in experiment diabetic neuropathy: (a) reduced survival of SCs due to down‐expressed neuritin contributed to diabetic neuropathy; (b) exogenous neuritin treatment ameliorated survivability of diabetic SCs through enhancing the Bcl‐2 level and depressing the caspase‐3 activity; and (c) increased viable SCs associated with neuritin pre‐treatment improved neurite outgrowth of co‐cultured DRG neurons from diabetic rats, most likely through increased production of NGF. Therefore, our study may provide a new mechanism and a new treatment for diabetic neuropathy. The detailed mechanism by which neuritin was down‐expressed in diabetic or hyperglycaemic conditions was not explored in this study. We did not find significant osmotic effects of high glucose on these SCs (mannitol control, data not shown). Insulin‐like growth factor‐1 (IGF‐1, not measured in this study) maybe one mediator since we observed that IGF‐1 levels reduced in experimental diabetes^19^, ^27^ and, in vitro, exogenous IGF‐1 stimulated neuritin expression in SCs.^24^ It is unclear what the relationship is between neuritin and other hyperglycaemia‐induced factors leading to diabetic neuropathy: a deficiency of other neurotrophins,^17^, ^18^, ^19^ enhanced polyol pathway activity,^5^, ^6^ increased non‐enzymatic glycation^7^ and augmented oxidative stress among others.

From regenerative or rehabilitative perspective, a deficit of neurotrophic factors affecting SCs is a very important cause of diabetic neuropathy. In this respect, our study provided evidence of a novel neurotrophic factor—neuritin deficit in the development of experimental diabetic neuropathy. Neuritin replenishment may be applied to practical in vivo treatment for diabetic neuropathy, if metabolism of neuritin is fully understood.

## CONFLICT OF INTEREST

The authors confirm that there are no conflicts of interest.

## AUTHOR CONTRIBUTIONS

**Chunhong Xi:** Conceptualization (lead); data curation (lead); formal analysis (lead); investigation (lead); methodology (lead); resources (supporting); software (equal); validation (equal); visualization (equal); writing‐original draft (equal). **Yingduan Zhang:** Conceptualization (lead); data curation (lead); formal analysis (lead); investigation (lead); methodology (lead); validation (equal); visualization (equal); writing‐original draft (equal). **Mei Yan:** Conceptualization (lead); data curation (lead); formal analysis (lead); investigation (lead); methodology (lead); software (equal); validation (equal); visualization (equal); writing‐original draft (equal). **Qing Lv:** Data curation (equal); formal analysis (equal); methodology (equal); project administration (equal); resources (supporting). **Huan Lu:** Data curation (equal); formal analysis (equal); investigation (supporting); methodology (supporting); project administration (supporting); resources (supporting). **Jun Zhou:** Data curation (equal); formal analysis (equal); investigation (supporting); methodology (supporting); project administration (supporting); resources (supporting). **Yucheng Wang:** Formal analysis (equal); investigation (equal); methodology (equal); project administration (supporting); resources (supporting); software (supporting). **Jianbo Li:** Conceptualization (lead); data curation (equal); formal analysis (lead); funding acquisition (lead); investigation (lead); methodology (equal); project administration (lead); resources (equal); software (equal); supervision (lead); validation (equal); visualization (equal); writing‐original draft (equal); writing‐review and editing (lead).

## Data Availability

Data were available and presented in the main manuscript.
